# Cellulose Nanofibril Based-Aerogel Microreactors: A High Efficiency and Easy Recoverable W/O/W Membrane Separation System

**DOI:** 10.1038/srep40096

**Published:** 2017-01-06

**Authors:** Fang Zhang, Hao Ren, Jing Dou, Guolin Tong, Yulin Deng

**Affiliations:** 1 Jiangsu Provincial Key Lab of Pulp and Paper Science and Technology, Nanjing Forestry University, 159 LongPan Road, NanJing, JiangSu Province 210037, P.R. China; 2 School of Chemical & Biomolecular Engineering and RBI, Georgia Institute of Technology, 500 10th Street N.W., Atlanta, Georgia 30332-0620, United States

## Abstract

Hereby we report a novel cellulose nanofirbril aerogel-based W/O/W microreactor system that can be used for fast and high efficient molecule or ions extraction and separation. The ultra-light cellulose nanofibril based aerogel microspheres with high porous structure and water storage capacity were prepared. The aerogel microspheres that were saturated with stripping solution were dispersed in an oil phase to form a stable water-in-oil (W/O) suspension. This suspension was then dispersed in large amount of external waste water to form W/O/W microreactor system. Similar to a conventional emulsion liquid membrane (ELM), the molecules or ions in external water can quickly transport to the internal water phase. However, the microreactor is also significantly different from traditional ELM: the water saturated nanocellulose cellulose aerogel microspheres can be easily removed by filtration or centrifugation after extraction reaction. The condensed materials in the filtrated aerogel particles can be squeezed and washed out and aerogel microspheres can be reused. This novel process overcomes the key barrier step of demulsification in traditional ELM process. Our experimental indicates the novel microreactor was able to extract 93% phenol and 82% Cu^2+^ from external water phase in a few minutes, suggesting its great potential for industrial applications.

Aerogel is a type of intriguing material prepared by replacing the liquid solvent in a gel with air and at the same time keeping the network structure. Cellulose is the most abundant biopolymer on the Earth. Moreover, it is safe, stable, nontoxic and biodegradable in nature. Nano cellulose based aerogel promises not only a very low density and high porosity which is typical in aerogels, but also a high strength and ductility compared with inorganic or polymeric aerogels^1^. Due to these advantages, the cellulose nanofibril (CNF) aerogel has received much attention in many applications, especially as a carrier in selective separation or as scaffold material and carriers for drug delivery in medicine^2^,^3^. Zhang *et al*. reported that cellulose nanofibril aerogel crosslinked by polyamide-epichlorohydrin resin showed very robust and fast shape recovery properties in water^4^. Also the functional crosslinked CNF aerogel with good mechanical properties can be directly used for adsorption contaminates in water and regenerated just by hand washing^5^,^6^.

The aerogel microsphere has many unique properties compared with those bulk cellulose materials, which can be used in various applications, such as in medical field^7^,^8^,^9^. The dimension and textural properties of the microspheres are key factors in some application such as *in-vivo* drug delivery and tissue engineering. Most routes to producing the aerogel microspheres were based on sol-gel formation, emulsion process, ambient pressure drying and supercritical extraction technique^10^. Cai *et al*. successfully fabricated cellulose nanofibril aerogel microspheres by using spray-freeze-drying method^11^. Diameter of most CNF aerogel microspheres is in the range of 60–120 μm. It has been reported that aqueous TEMPO ((2,2,6,6-Tetramethylpiperidin-1-yl)oxyl) oxidized cellulose nanofibril (TOCN) suspensions consist of anion charges, thus they are highly homogeneous. The process of using an ultrasonic nebulizer to create TOCN tiny suspension through high-frequency oscillation is a very effective method for preparing small and uniform cellulose-water droplets. In medicine, an ultrasonic nebulizer is a device used to make liquid medicine into fine droplets, such as in aerosol or mist form which is very small droplets with a very narrow size distribution. As a consequence, the particles travel into the whole alveoli and the nebulized medication solution is able to be sufficiently absorbed by the human respiratory tract, resulting in the best therapeutic effects^12^.

Emulsion liquid membrane (ELM) is a promising technique for separation of contaminants such as metal ions, weak acids/bases, inorganic species and hydrocarbons due to the high interfacial area for mass transfer, the ability to remove and to concentrate selectively or collectively, and the requirement of only small quantities of organic solvent^13^. However, commercially applications of ELM is still not available mainly because of difficulty in demulsification and reuse of the oil phases. Emulsion instability is another issue which may result in the membrane leakage, coalescence, and emulsion swelling. The lack of stability of the emulsion globules will decrease extraction efficiency. On the other hand, an overly stable emulsion causes problems during its settling and demulsification after it extracts the pollutants. Also, high energy must be supplied to produce stable mixtures in each stage^14^. In order to solve instability problem and de-emulsification of ELM, we report in this study using cellulose nanofibril based-aerogel micropsheres as the microcollector. There are several advantages of using aerogel microspheres as a new membrane separation system: (a) the small aerogel diameter tending to have rapid extraction. Li *et al*. suggested that, for ELM system, the optimized emulsion droplets should have a size between 0.3–10 um with good stability^15^; (b) the highly porous and super-water-absorbent TOCN aerogel microspheres can accommodate large amount of internal phase and allow the proper extraction; (c) the most important that crosslinked aerogel microspheres can be easily separated and regenerated after extracting step, which overcomes the key barrier in the ELM separation.

In this study, a facile approach was reported for preparation of TOCN aerogel microspheres using an atomization-freeze-drying method. The ultralight (~2.1 mg cm^−3^) micropsheres possess small and uniform particle size (mostly within 2–7 um), and high water absorbency (~120 g g^−1^). The extraction applications of the membrane based on aerogel microspheres could be changed by varying stripping agent in the microspheres and carriers in organic phases. The results showed that 93% of phenol and 82% of Cu^2+^ can be extracted from aqueous solution by using TOCN aerogel microsphere based microreactor. Moreover, the used microspheres could be easily regenerated by simply filtration, squeezing and washing. The regenerated microreactor system exhibits excellent reuse ability. All these demonstrated that cellulose nanofibril aerogel microsphere based membrane could be served as a new extraction tool and has great potential for industrial applications.

## Results

**Figure 1:**
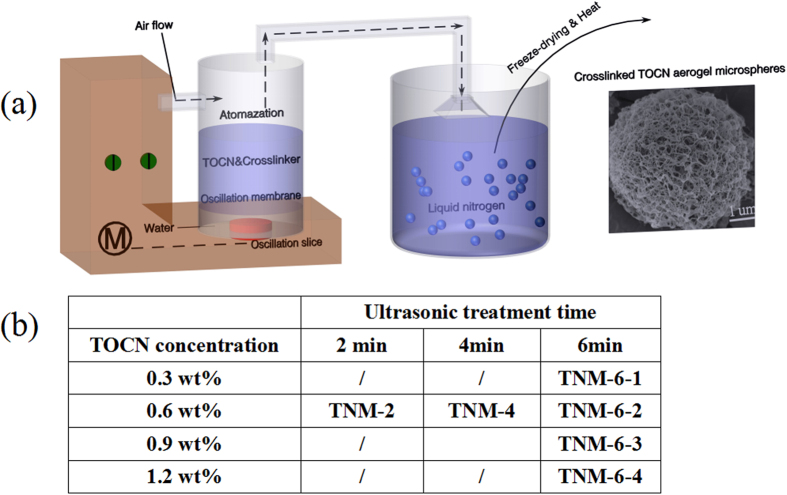
(**a**) Schematic illustration of ultrasonic atomization and freeze-drying method for fabricating the crosslinked TOCN aerogel microspheres. (**b**) The nomenclature of TOCN aerogel micropsheres.

Softwood pulp fibers with diameters between 20 and 50 um were used as the starting material to prepare TOCN. After TEMPO-oxidation, the carboxyl group content of the cellulose was 1.83 mmol g^−1^, which was measured by conductance titration method. Afterwards, the TEMPO oxidized cellulose suspension was subject to the mechanical defibrillation by using ultrasonic processor to obtain TOCNs. When it was dispersed in water, TOCN did not flocculate, but instead became a homogeneous mixture. It was found that the ultrasonic atomization method could not only produce small and uniform droplets but also avoid the unstable atomization problems. Schematic illustration of ultrasonic atomization and freeze-drying method for fabricating the crosslinked TOCN aerogel microspheres were shown in Fig. 1(a). The atomized TOCN aerosols were directly sprayed into liquid nitrogen. The frozen spherical samples were freeze-dried to obtain aerogel microspheres. The dried aerogel microspheres were cured in a vacuum oven at 120 °C for 3 h to achieve sufficient covalent crosslinking. Polyamide-epichlorohydrin resin was widely used as a wet strength agent in paper industry. The crosslinker of the resin can form both self-crosslinking and external crosslinking with cellulose, which can also be used to increase wet strength of aerogel microspheres.

**Figure 2:**
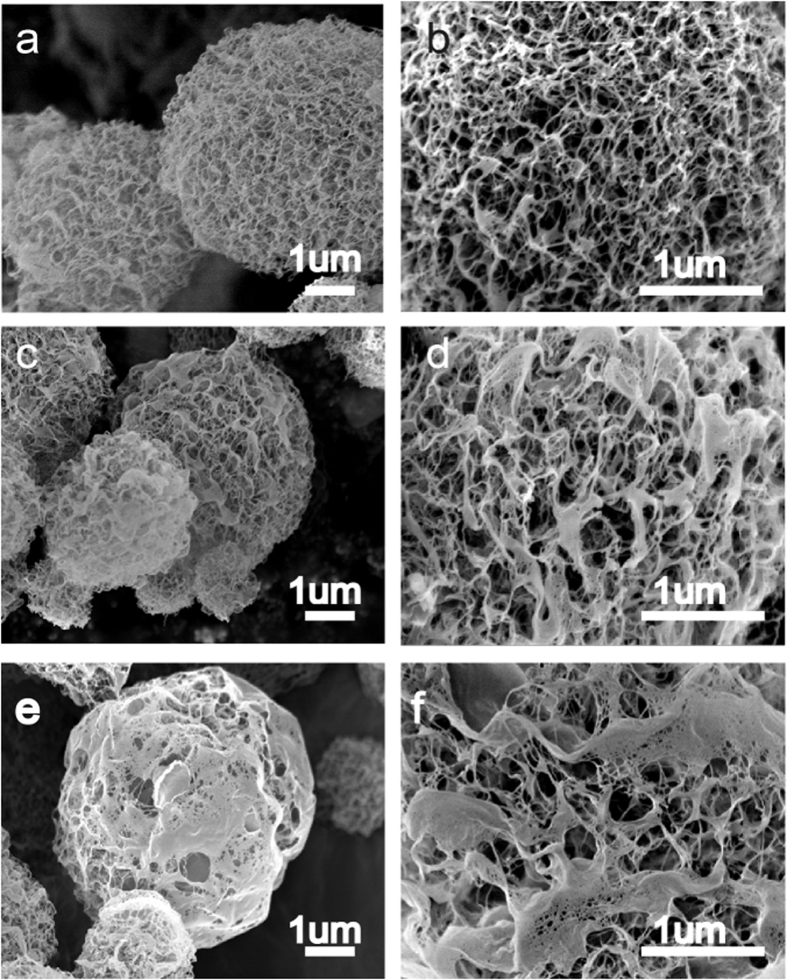
(**a**,**b**) SEM images of TNM-6-2; (**c**,**d**) SEM images of TNM-4; (**e**,**f**) SEM images of TNM-2.

The morphology changing in the TOCN aerogel microspheres due to the atomization of TOCN suspensions with different ultrasonic treatment time during the preparation of TOCN was evaluated through SEM pictures (Fig. 2). Three samples of TOCN aerogel microspheres were produced using the same TEMPO-mediated oxidation pre-treatment but different extents of subsequent mechanical treatment in an ultrasonic processor. From Fig. 2(a), it is clear that the TOCN aerogel microspheres are highly porous, and the morphology of the aerogel appears to be a homogeneous network. Under the higher magnification in Fig. 2(b), it could be found that diameters of some nanofibril bundles are approximately dozens of nanometers. The pore size from Fig. 2(b) is typically in the range of nanometers to micrometers, which is significantly more homogeneous than those shown in Fig. 2(c–f). By comparing the SEM images of (c) (d) and (e) (f), it is obvious that when the ultrasonic treated time decreased, the structure of microspheres became more compact. Some cellulose bundles are even not disintegrated to nano size if a short time of ultrasound treatment time is adopted. The harsh disintegration conditions for TOCN were adopted in this study. It is obvious that when the ultrasound time was increased during disintegration process, the morphology became more porous.

**Figure 3:**
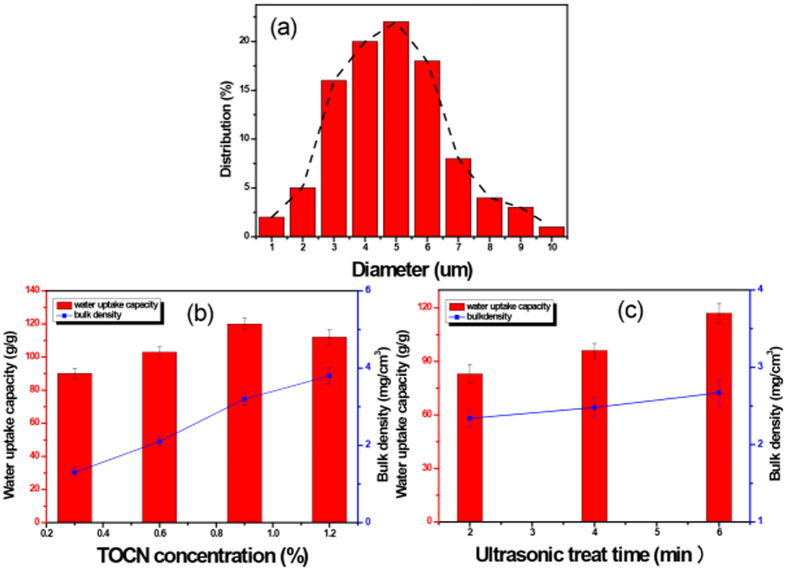
(**a**) Particle size distribution of TNM-6-2. Physical properties of (**b**) TNM-6-1. TNM-6-2, TNM-6-3, TNM-6-4; (**c**) TNM-2,TNM-4, TNM-6-2.

The size of TOCN aerogel microspheres was investigated by SEM. The particle size was determined by the droplet size produced from the ultrasonic nebulizer. Diameters of the microspheres were within 1–10 um with a majority of 2–7 μm, which is shown in Fig. 3(a). Figure 3(b,c) gathers physical properties of the TOCN aerogel microspheres. By comparing different samples in Fig. 3(b), the TOCN aerogel microspheres density is almost linearly proportional to dispersion concentrations. Although the bulk density of TNM-6-4 is almost 2 times of TNM-6-1, their water uptake capacities are very similar (95–118 g g^−1^). The superabsorbent properties of TOCN aerogel microspheres depend not only on the hydrophilic nature of TOCN but also largely on the storability of the pores inside. When the aerogel microsphere was dipped into an aqueous medium, the water easily diffused into the aerogel matrix through the micro pores, and was then entrapped in the aerogel microspheres. By comparing different samples in Fig. 3(c), it has been found that although the bulk density of the aerogel microspheres is quite similar, water absorption capacities of these absorbents varies widely from 80 to 120 g g^−1^. The phenomenon was presumably caused by different pore structures which could be seen in Fig. 2. It is obviously that the higher porous structure of TNM-3 could hold larger amount of water compared to other samples.

**Figure 4:**
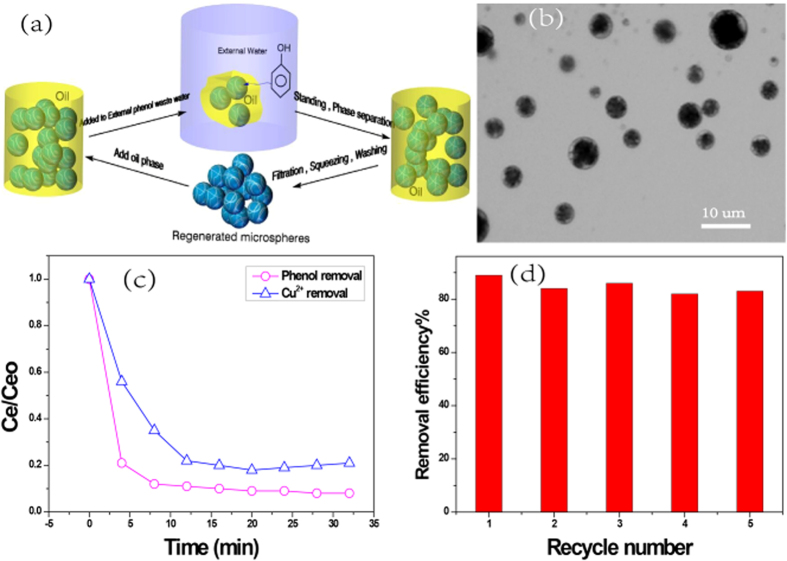
(**a**) Schematic illustration of cellulose nanofibril based-aerogel as W/O/W membrane microreactor for extraction and separation. (**b**) Optical microscope image of saturated aerogel microspheres dispersed in Kerosene-silicone oil. (**c**) Extraction of phenol using NaOH saturated TNM-6-2 based membrane, NaOH = 0.8 wt%, oil:saturated aerogel = 1.5:1 (v/v), membrane phase: external phase = 1:2 (v/v), Span80 = 2 wt% based oil; Extraction of Cu^2+^ using EDTA saturated TNM-6-2 based membrane, EDTA-2Na = 1 wt%, oil:saturated aerogel = 1.5:1 (v/v), membrane phase: external phase = 1:2 (v/v), Span80 = 2 wt% based oil, Metral^®^ 84 H = 10 vol% based oil. (**d**) Phenol extraction efficiency as a function of the repeat cycle using regenerated TNM-6-2.

It is believed that the small droplet diameter of microsphere is a key criterion that will provide a more stable suspension and higher mass transfer rate when it is used as W/O/W microreactor separation system. To establish an effective and stable microsphere based microreactor system, an organic solvent that can disperse the water saturated aerogel particles must be founded. The oil density and viscosity could play an important role. There are also some other requirements on selection of the solvent: low solubility in the internal and external aqueous phase, compatibility with the surfactant, moderate viscosity (not too low as to compromise membrane stability), having a density that is sufficiently different from the aqueous phase^16^. Kerosene was chosen in this study because it is a low cost and most widely used solvent in ELM. However, it was quickly found that the saturated aerogel microspheres could not be well dispersed in kerosene even if a surfactant Span 80 was used. Instead, we found that aerogel microspheres could be well dispersed in a mixture of kerosene-silicone oil (volume ratio of 1:1), as shown in Fig. 4(b), and they could also be easily separated from the external aqueous phase by simply filtration after extraction.

Schematic illustration of cellulose nanofibril based-aerogel as W/O/W membrane microreactor for extraction and separation is shown in Fig. 4(a), and the detailed technical process is shown in Supplementary Fig. S1. As can be seen from Fig. 4(c), more than 93% phenol and 82% Cu^2+^ were removed from external phase by aerogel based W/O/W microseparator system within a few minutes. For phenol extraction, phenol molecule was transferred through the organic membrane and stripped by NaOH due to the formation of phenol-Na in the aerogel micropsheres. Surfactant (Span80) can be used to lower the surface tension between oil and saturated aerogel micropsheres, which therefore increases the suspension stability so the removal efficiency in the extraction process^17^. On the other hand, as more surfactants are added, thickness of emulsion globules increases, which causes higher mass transfer resistance and inversely decreases the removal efficiency. It was found that 2 wt% Span80 (based on oil) gave the best extraction efficiency among the tests conducted in this study. For Cu^2+^ extraction experiments, the carrier Metral^®^ 84 H was added in the organic phase in order to transfer Cu^2+^ from external phase to aerogel phase. EDTA is one of the most widely used stripping agents. The internal aerogel phase consisted of EDTA-2Na can be used to de-extraction Cu^2+^ ions from Metral 84 H to aerogel microreactor, because Cu-EDTA chelated anions could not transport back through the organic phase. All these demonstrated that the cellulose nanofibril aerogel microsphere based membrane has a potential for fast and high efficient extraction or separation for variety materials.

Although ELM method is very effective and has been successfully studied for a few decades, its commercial applications on removing waste pollutants have been limited due to the difficulty in demulsification after extraction. All physical demulsification methods such as centrifugation and high-voltage electric field were expensive, low efficiency and energy-consuming^18^. Chemical demulsification methods are usually restricted in ELM process because the difficulty in reusing the oil phose. In our aerogel microsphere based separation system, both TOCN aerogel microspheres and the organic phase could be recovered and reused by a simply filtration process. The concentrated materials, such as Cu^2+^ ions and phenol in the aerogel microspheres could be easily squeezed and washed out, and the aerogel microspheres can be completely reused. Experimentally, the recovered microspheres were recovered, washed and reused for five times in this study. It was found that the crosslinked aerogel microspheres could be well-kept during the regeneration and isolation processes. The morphology of regenerated aerogel microspheres after 5 regeneration circles can be seen in Supplementary Fig. S2. As can be seen in Fig. 4(d), after five regeneration cycles, the aerogel-based microreactors still shows a high effective extraction efficiency, which suggests high reusability of cellulose nanofibril aerogel as a microreactor in W/O/W membrane separation system.

## Conclusion

In summary, we successfully fabricated cellulose nanofibril aerogel microspheres by using a ultrasonic nebulizer. These aerogel microspheres mostly ranging from 2 um to 7 μm were highly porous and ultra-lightweight. Water uptake tests showed that more than 120 g g^−1^ water can be absorbed. The structure of microsphere aerogel particles was changed by varying the concentration of TOCN suspension and the isolation intensity during the preparation process. Through covalently crosslinking, the robust aerogel micropsheres are stable even in a harsh environment. All these properties showed promising potential for various applications, especially as a W/O/W suspension microreactors for ions extraction from water. The saturated aerogel microspheres can be well dispersed in the organic phase to form a stable suspension by simple low shear stirring. Also, the aerogel microspheres can be easily separated by filtration and reused in a new separation system without a complicated demulsification process. All these demonstrated that the natural, green aerogel microspheres have great potential in separation and extraction applications.

## Experimental

### Preparation of cellulose nanofibril aerogel microspheres

Commercial fully bleached softwood kraft pulp was used as the starting material to prepare TEMPO-oxidized cellulose nanofibrils. Briefly, cellulose fibers were suspended in water containing TEMPO and sodium bromide (0.016 and 0.16 gram per gram of cellulose respectively). The oxidation was started by adding a desired amount of NaClO (20 mmol NaClO per gram of cellulose). pH was maintained at 10.5 by adding 2 mol L^−1^ NaOH solution. After 2 h, the obtained cellulose was washed with distilled water, and the obtained cellulose fiber suspensions were diluted to the desired concentration and then subjected to the mechanical defibrillation process by using a probe-type ultrasonic processor (Ultrasonics FS-300) to produce TOCN. Then, a weighted amount of crosslinker (Polyamide-epichlorohydrin resin) was added into the TOCN suspensions with mechanical stirring. About 150 ml of nanofibril suspensions were transferred into a cup that is impacted by ultrasonic waves of the medical ultrasonic nebulizer (Yuyue, 420AI, Power: 1.7 MHZ ± 10%), the TOCN suspensions were atomized and then blown into liquid nitrogen for instant freezing followed by freeze-drying at −50 °C for 12 h (Freeze-dryer: Scientz-12N). In order to obtain the crosslinked aerogel spheres, dried samples were cured in a vacuum oven at 120 °C for 3 h. In order to make a comparison of the properties, samples prepared with different conditions were shown in Fig. 1(b).

### Characterization

Scanning electron microscopy (SEM, JSM-7600F) was used for morphological characterization. Bulk density was determined by transferring a known quantity of microspheres to a 100 mL measuring cylinder and tapping it 3 times at 2 s intervals. The bulk density was obtained through dividing the weight of the sample by the final volume of the sample. Water uptake capacity was determined by the gravimetric method. About 0.5 g of dried sample was weighed and then soaked in water until the weight remained constant. The wetted samples were wiped with a piece of blotting paper and immediately weighed, so that the water uptake capacity can be determined by their increased weight. All tests of water uptake capacity and bulk density of the microspheres were repeated 3 times. The absorption capacities on NaOH (0.8 wt%) solution as well as the EDTA solution (1 wt%) were measured according to the method above. The results indicated that about 93 g of 0.8 wt% NaOH and 101 g 1 wt% EDTA solution were absorbed by 1 g TNM-6-2 respectively. Particle size of TOCN aerogel microspheres was determined by averaging more than 100 particles using several images obtained from scanning electron microscopy (SEM, JSM-7600F).

### Pollutant removal studies

Phenol was extracted from external aqueous solution into the nanocellulose aerogel based microreactor. The oil membrane phase was prepared by mixing surfactant (Span 80), kerosene-silicone oil mixture and TOCN aerogel microspheres (TNM-6-2) saturated with NaOH solution (0.8 wt%) under mechanical stirring (1500 rpm min^−1^) for 30 min. The membrane was then poured into the phenol solution (100 mg L^−1^) and stirred at a low speed (200 rpm). UV-vis Spectrophotometer (UV757CRT/PC) was used to measure the concentration of phenol in the separated external phase (wavelength at 287 nm). Copper (100 mg L^−1^) solution was prepared by dissolving desired amount of CuSO_4_ 5H_2_O in deionised water. The aerogel microspheres (TNM-6-2) were first saturated with 1 wt% EDTA solution (internal stripping agent), then mixed with a mixture of silicone oil-kerosene that contains 10 vol% carrier (Mextral^®^ 84 H) and 2 wt% emulsifier (Span 80) under a mechanical stirring at 1500 rpm for 30 min. The membrane was then poured into the Cu^2+^ solution and stirred at a low speed (200 rpm). The concentration of Cu^2+^ was analyzed by an atomic absorption spectrophotometer (TAS-990) at 324 nm. Once the extraction step was finished, the membrane and external phase was separated by settling. The oil phase containing water-saturated aerogel microspheres was filtrated and the nanocellulose microspheres were collected. The filtrate containing organic oil, emulsifier and carrier was directly reused. The filtered water-saturated aerogel microspheres were regenerated by squeezing out of the phenol-Na solution and reused for new batch of experiment.

## Additional Information

**How to cite this article**: Zhang, F. *et al*. Cellulose Nanofibril Based-Aerogel Microreactors: A High Efficiency and Easy Recoverable W/O/W Membrane Separation System. *Sci. Rep.* **7**, 40096; doi: 10.1038/srep40096 (2017).

**Publisher's note:** Springer Nature remains neutral with regard to jurisdictional claims in published maps and institutional affiliations.

## Supplementary Material

Supplementary Information
