# Supplementary material for: Cellulose Nanofibril Based-Aerogel Microreactors: A High Efficiency and Easy Recoverable W/O/W Membrane Separation System
Source: Sci Rep. 2017 Jan 6;7:40096. doi: 10.1038/srep40096 (PMC5216329; doi:10.1038/srep40096)
Supplement: Supplementary Information [file srep40096-s1.pdf]

Cellulose Nanofibril Based-Aerogel Microreactors: A High Efficiency and Easy Recoverable W/O/W Membrane Separation System

Fang Zhang[1,2], Hao Ren[1], Jing Dou[1], Guolin Tong[1,*], and Yulin Deng[2,*]

[1]Jiangsu Provincial Key Lab of Pulp and Paper Science and Technology, Nanjing Forestry University, 159 LongPan Road, NanJing, JiangSu Province 210037, P. R. China
[2]School of Chemical & Biomolecular Engineering and RBI, Georgia Institute of Technology, 500 10th Street N.W., Atlanta, Georgia 30332-0620, United States
*yulin.deng@rbi.gatech.edu

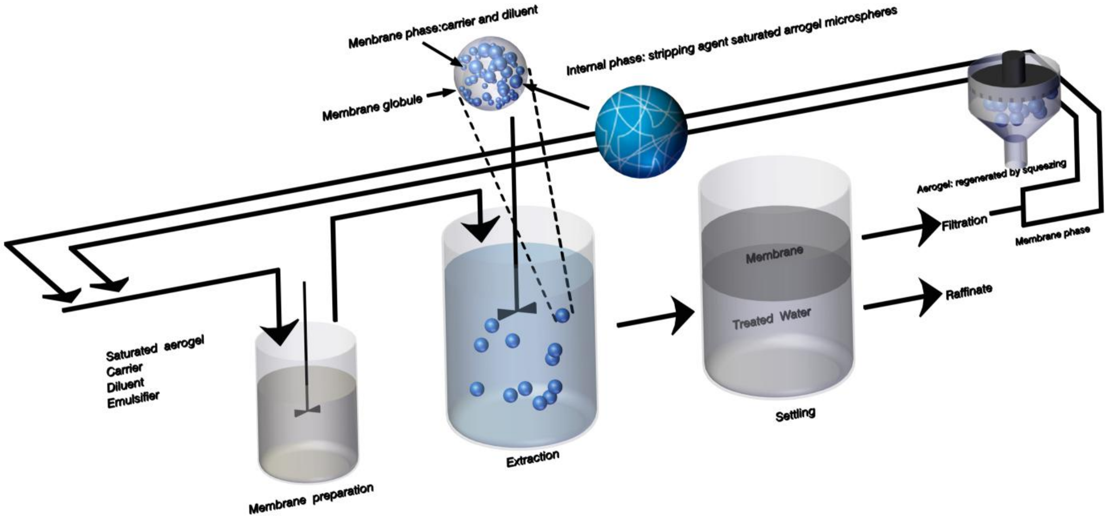

Supplementary Figure S1. Schematic illustration of cellulose nanofibril aerogel microspheres based W/O/W extraction process.

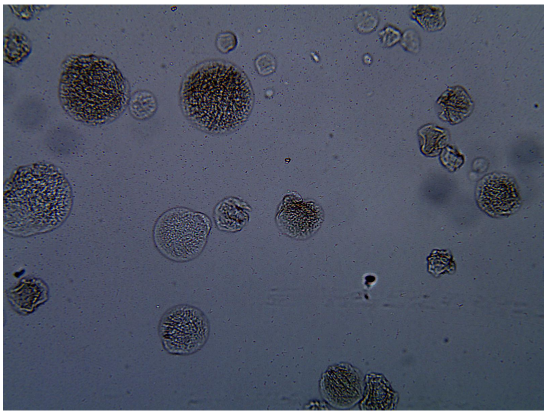

Supplementary Figure S2. The morphology of regenerated aerogel microspheres after 5 regeneration circles.
